# Microsatellite polymorphisms associated with human behavioural and psychological phenotypes including a gene-environment interaction

**DOI:** 10.1186/s12881-017-0374-y

**Published:** 2017-02-03

**Authors:** Andrew T. M. Bagshaw, L. John Horwood, David M. Fergusson, Neil J. Gemmell, Martin A. Kennedy

**Affiliations:** 1 Department of Pathology, University of Otago, Christchurch, PO Box 4345, Christchurch, New Zealand; 2 Department of Psychological Medicine, University of Otago, Christchurch, New Zealand; 3 Department of Anatomy, University of Otago, Dunedin, New Zealand; 4 Gravida - National Centre for Growth and Development, University of Otago, Dunedin, New Zealand

**Keywords:** *TBR1*, Androgen receptor, *AP2-β*, Microsatellite, STR, Antisocial, Behaviour, Pregnancy, Smoking, Gene-environment interaction

## Abstract

**Background:**

The genetic and environmental influences on human personality and behaviour are a complex matter of ongoing debate. Accumulating evidence indicates that short tandem repeats (STRs) in regulatory regions are good candidates to explain heritability not accessed by genome-wide association studies.

**Methods:**

We tested for associations between the genotypes of four selected repeats and 18 traits relating to personality, behaviour, cognitive ability and mental health in a well-studied longitudinal birth cohort (*n* = 458-589) using one way analysis of variance. The repeats were a highly conserved poly-AC microsatellite in the upstream promoter region of the T-box brain 1 (*TBR1*) gene and three previously studied STRs in the activating enhancer-binding protein 2-beta (*AP2-β*) and androgen receptor (*AR*) genes. Where significance was found we used multiple regression to assess the influence of confounding factors.

**Results:**

Carriers of the shorter, most common, allele of the *AR* gene’s GGN microsatellite polymorphism had fewer anxiety-related symptoms, which was consistent with previous studies, but in our study this was not significant following Bonferroni correction. No associations with two repeats in the *AP2-β* gene withstood this correction. A novel finding was that carriers of the minor allele of the *TBR1* AC microsatellite were at higher risk of conduct problems in childhood at age 7-9 (*p* = 0.0007, which did pass Bonferroni correction). Including maternal smoking during pregnancy (MSDP) in models controlling for potentially confounding influences showed that an interaction between *TBR1* genotype and MSDP was a significant predictor of conduct problems in childhood and adolescence (*p* < 0.001), and of self-reported criminal behaviour up to age 25 years (*p* ≤ 0.02). This interaction remained significant after controlling for possible confounders including maternal age at birth, socio-economic status and education, and offspring birth weight.

**Conclusions:**

The potential functional importance of the *TBR1* gene’s promoter microsatellite deserves further investigation. Our results suggest that it participates in a gene-environment interaction with MDSP and antisocial behaviour. However, previous evidence that mothers who smoke during pregnancy carry genes for antisocial behaviour suggests that epistasis may influence the interaction.

**Electronic supplementary material:**

The online version of this article (doi:10.1186/s12881-017-0374-y) contains supplementary material, which is available to authorized users.

## Background

Twin studies indicate that personality and behavioural traits including antisocial behaviour have a strong genetic component [1–4]. However, similarly to many other complex traits, the sources of their heritability have not been uncovered to a substantial degree by genome-wide association studies (GWAS) [3, 5–9]. Accumulating evidence suggests that heritability not so far accessed by GWAS resides partly in under-studied forms of genetic variation including length polymorphism in arrays of short tandem repeat (STR) sequences, otherwise known as microsatellites [10–13]. This is not adequately represented in GWAS because the frequency and diversity of microsatellite polymorphism are much higher than those of single nucleotide polymorphism (SNP) [11, 14].

Many species including bacteria and yeasts are known to have harnessed the high mutability of microsatellites for regulatory purposes, and there is evidence that microsatellite functionality is far more widespread in the human genome than has traditionally been appreciated [11–13, 15–18]. For example a recent study of expression quantitative trait loci estimated that at least 10–15% of the heritability in human gene expression levels attributable to common variants in *cis* is due to microsatellite polymorphism [12]. Candidate-gene association studies for a growing number of human regulatory microsatellites have been well replicated [19–24], and mechanisms by which microsatellites function, including modification of spacing between adjacent transcription factors and other promoter elements [25–27], regulation of splicing [22, 28, 29], adoption of structural variants such as Z-DNA [30–32], and modification of epigenetic signals [12] are increasingly well understood. Despite this body of evidence, examples of phenotypic associations with microsatellites remain isolated. This is mainly due to difficulties genotyping large numbers of loci, and while methods have recently been developed that will facilitate microsatellite-based association studies comparable in scale to contemporary SNP-based GWAS, these still require extensive sequencing and/or capture probe synthesis [11, 33].

Previous work by our group has attempted to identify likely functional microsatellites by investigating their conservation among mammalian species [34, 35], and links between microsatellites and several neurological disorders and behavioural phenotypes suggest that they are particularly likely to be functionally important when associated with brain-related genes [13, 36]. These considerations motivated us to investigate associations between human behavioural traits and a previously unstudied AC_12-13_ microsatellite in the upstream promoter region of the T-Box Brain 1 (*TBR1*) gene, which is in the top 2.5% of human microsatellites by level of conservation [34]. *TBR1* is a member of the set of regulatory genes involved in the genesis and fate determination of glutamatergic neurons [37–39]. As a transcription factor it regulates as many as 124 other genes [40, 41] including *GRIN2B*, which has been implicated in attention deficits in children [42], cognitive performance [43], neuroticism [44] and smoking [45] among other psychiatric and behavioural phenotypes [46]. *TBR1* haploinsufficiency in mice results in defective axonal projections of amygdalar neurons and the impairment of social interaction, ultrasonic vocalization, associative memory and cognitive flexibility [40]. In humans, deletion of chromosomal regions including *TBR1* is associated with intellectual disability [47], and mutations of the gene have been linked to autism spectrum disorder [48, 49]. *TBR1* was also recently distinguished as the highest scoring locus associated with educational attainment in a study of more than 300,000 individuals [50].

We also sought to replicate and/or expand on previous studies showing associations between psychological and/or behavioural phenotypes and three other tandem repeats. The GGN microsatellite in exon 1 of the androgen receptor (*AR*) gene encodes a poly-glycine stretch associated with receptor responsiveness [51, 52]. It has been less studied than the CAG microsatellite in the same exon, which is associated with diseases including spinobulbar muscular atrophy [53], but its common length variants have been linked to externalizing behaviours including conduct disorder [54] and the personality traits self-transcendence [24, 55], aggression and impulsivity [56]. Two intronic repeats in the *AP-2β* transcription factor gene have also been studied relatively little. A minisatellite in its first intron has been associated with the impulsivity-linked phenotypes alcohol dependence [57] and late auditory-evoked potentials [58]. A CAAA microsatellite in the second intron of the same gene has been linked to reduced MAOB activity, which is also related to impulsivity [59], late auditory-evoked potentials [58], anxiety-related measures [60] and the personality traits self-transcendence and spiritual acceptance [61]. The *AP-2β* transcription factor functions in neural development and influences brain monoaminergic systems by regulating target genes [62, 63].

The initial aim of this study was to assess the effects on human traits relating to personality, behaviour, cognitive ability and mental health of our selected repeat polymorphisms. The most significant associations we found were between the *TBR1* microsatellite and antisocial behaviour. Following this, recent interest by two of our authors (LJH and DMF) in maternal smoking during pregnancy (MSDP) and its effects on offspring behaviour [64, 65], led us, in conjunction with evidence showing expression of *TBR1* in the human embryonic neocortex [37], and linking the gene to responses to prenatal cannabis exposure [66], and maternal illness [67], to the additional hypothesis that MSDP may modify the effects of *TBR1* microsatellite genotype.

Our test subjects were from a well-studied birth cohort, the Christchurch Health and Development Study (CHDS). The CHDS is a longitudinal study of 1265 children born in Christchurch, New Zealand who have been studied on 22 occasions from birth to age 30 [68, 69], 570 of whom were included in the work presented here. As part of this study data were gathered on: a) MSDP; b) measures of antisocial behaviour assessed from early childhood to mature adulthood by a wide range of methods; c) potentially confounding social and contextual factors.

## Methods

### Sample

The sample group was from the CHDS birth cohort of 1265 children born in the Christchurch (New Zealand) urban region in mid-1977 who have been studied at birth, 4 months, 1 year, annually to age 16 years, and again at ages 18, 21, 25 and 30 years [68, 69]. At age 28–30 surviving cohort members were approached to provide a blood sample for genetic analysis. Those unwilling or unable to provide a blood sample were asked for a saliva sample instead. Of those approached 916 (91%) agreed to provide a DNA sample (90% bloods, 10% saliva), and 679 (74%), 537 (59%), 702 (77%) and 684 (75%) were successfully genotyped for TBR1, AR, AP2-β AAAC and AP2-β minisatellite respectively. The remainder could not be genotyped despite repeated attempts, suggesting DNA sample degradation. Since preliminary analysis suggested the presence of ethnic stratification with respect to the distribution of both *TBR1* and *AP2-β* CAAA genotypes, the primary analysis was restricted to those of sole European ancestry defined on the basis of parental reports of ethnic ancestry; the non-European sample comprised 14.1% of the cohort. The final analysis samples with data on both genotype and at least one outcome were respectively 570, 459, 589 and 571 for the four genes. Actual sample sizes vary slightly from these Ns depending on the pattern of missing data for each outcome. The process of DNA collection and preparation has been described elsewhere [70].

### Genotyping

PCR primers and genomic locations of each of the four repeats are listed in Table 1. All forward primers were 5′-labelled with compatible fluorophores (Applied Biosystems Foster City, CA, USA). Polymerase chain reaction conditions were as follows: initial 2-min denaturing step at 95 °C, followed by 35 cycles of 94 °C for 45 s, 60 °C for 45 s and 72 °C for 45 s, and a final extension phase of 72 °C for 5 min. Reactions were performed in 10 μl volumes using PCR buffer with 1.75 mM MgCl_2_, ∼50 ng of genomic DNA, 500 nM of each primer, 200 μM of each dNTP and 0.5 units of Taq polymerase (Fisher Biotech Wembley, WA, Australia). Polymerase chain reaction products were assayed on an Applied Biosystems 3130xl genetic analyser, using GeneScan 500 LIZ (Applied Biosystems) as size standard. Results were analysed with GeneMapper v 4.0 software for Windows (Applied Biosystems). Analyses were restricted to homozygous major allele carriers vs other genotypes (explained below), and the copy numbers and frequencies of the most common alleles are shown in Table 1. All of these alleles were confirmed by sequencing five homozygotes of each genotype using an Applied Biosystems 3130xl genetic analyser (data not shown).

**Table 1:** Location, repeat motif, copy number, frequency of most common alleles, and PCR primers for each of the four repeats studied

Gene	Motif	Copies (freq)	GRCh38 coordinates	Primers (fwd/rev)
*TBR1*	AC	12 (0.82), 13 (0.18)	chr2:161,415,158-161,415,183	GCTTTTTCTCCCCTCCAGAT GGGAGACAGAGTGGAAGTGG
*AR*	GGN	23 (0.59), 24 (0.41)	chrX:67,546,497-67,546,572	CTCTTCACAGCCGAAGAAGG AGGCGACATTTCTGGAAGGA
*AP-2β*	GCGGACGAG	9 (0.22), 10 (0.61)	chr6:50,819,803-50,819,893	CTCCTGTGTGGGCATCTTTC CCAGACCATTCCGCTTAAAAG
*AP-2β*	CAAA	4 (0.4), 5 (0.59)	chr6:50,823,873-50,823,896	GGCAGCGGCAAGAAGTGGGT AGACAGAGCTGCCCTCTCCTG

### Phenotyping

An initial series of 18 measures was selected from the study database to examine for associations with the repeat variants. These measures spanned the following domains: child behaviour (conduct problems, attentional problems, anxiety/withdrawal); cognitive ability (child IQ, scholastic ability); adolescent personality (neuroticism, extroversion, novelty seeking) and self-esteem; and mental health symptoms in adolescence (somatisation, anxiety, phobic anxiety, obsessive-compulsive, depression, interpersonal sensitivity, psychoticism, paranoid ideation, hostility). A detailed description of these measures is provided in the Additional file 1. However, the principal results reported in this paper relate to exploration of a specific association between *TBR1* and childhood conduct problems at age 7–9, and the identification of a possible gene by environment interaction between *TBR1*, MSDP and the development of broader antisocial behaviours. The specific measures used in this component of the analysis are described in detail below.

### Measures of antisocial behaviour

Four measures of antisocial behaviour were selected from the database of the study to span the period from childhood to young adulthood.

#### Childhood conduct problems (7–9 years)

When sample members were aged 7, 8 and 9 years parental and teacher reports of the child’s tendencies to disruptive, oppositional and conduct disordered behaviours were obtained using an instrument that combined items from the Rutter [71] and Conners [72] parent and teacher questionnaires. The selected items spanned a range of behaviours relating to disobedience and defiance of authority, fits of temper and irritability, aggression or cruelty towards others, destruction of property, lying, stealing and other similar behaviours. Confirmatory factor analysis of the selected items for each source (parents, teachers) suggested that, in each case, the items could be scaled as unidimensional scales representing the extent of child conduct problems as reported by parents and teachers [73]. Scale scores representing the extent of disruptive, oppositional or conduct disordered behaviour at age 7, 8 and 9 years were created by summing parental and teacher item scores for each child at each age. These scales were then averaged over the 3-year period to provide an overall measure of the extent of conduct problems in middle childhood. The reliability of this scale, assessed using coefficient alpha, was 0.97. To simplify presentation in the present analysis the scale scores have been standardised to a mean of 100 and standard deviation of 10.

#### Adolescent conduct problems (15–16 years)

When participants were aged 15 and 16 years parental and self-report measures of the child’s tendencies to oppositional and conduct disordered behaviours were obtained. Parental reports were based on a selected series of items from the Revised Behaviour Problems Checklist [74]. Children were asked a series of questions relating to conduct/oppositional defiant behaviors derived from the Diagnostic Interview Schedule for Children [75]. In a similar fashion to the behavior reports in middle childhood, a total symptom score was constructed for each child at each age by summing the parent and self-report items. These symptom scores were then averaged over the 2 years to provide an overall measure of the child’s tendencies to conduct disordered and oppositional behaviours in adolescence. The reliability of this scale, assessed using coefficient alpha, was 0.87. In the present analysis the scale scores have been standardized to a mean of 100 and standard deviation of 10.

#### Self-reported property/violent offences (14–25 years)

At each assessment from age 15 to 25 years participants were questioned about their involvement in criminal offending since the previous assessment. At ages 15 and 16 questioning was conducted using the Self-Report Early Delinquency Scale [76]. At ages 18, 21 and 25 questioning was based on the Self-Report Delinquency Inventory [SDRI; 45], supplemented by additional custom-written survey items. This information was used to derive count measures of the number of self-reported property and/or violent offences committed in each year from age 15 to age 25. Property offences were defined to include theft, burglary, breaking and entering, vandalism, fire setting, and related offences; violent offences included assault, fighting, use of a weapon, threats of violence against a person, cruelty to animals and related offences. For the purposes of the present analyses, the number of offences committed in each year was summed over the period 15–25 years to create an overall score reflecting the total number of property or violent offences reported over the period from adolescence to young adulthood. To avoid problems with extreme outliers, the number of reported offences was truncated to a maximum of 500.

#### Self-reported arrests/convictions (16–25 years)

At ages 18, 21 and 25 years participants were questioned about their contacts with the police and criminal justice system for each 12 month period since the previous assessment. This information included details of any arrests or court convictions received in each interval. To provide an overall measure of the level of involvement with the criminal justice system in young adulthood, these report data were used to construct a count of the total number of arrests/convictions reported over the interval from age 16–25 years.

### Maternal smoking during pregnancy

At the time of the survey child’s birth, mothers were questioned about cigarette smoking during pregnancy, and specifically how much they smoked (cigs/day) in each trimester. These reports were averaged over the three trimesters to provide a measure of the average number of cigarettes smoked per day during pregnancy. In the analysis below this variable is used in two ways: (a) to define a dichotomous (smoker/non-smoker) measure of smoking during pregnancy for use in the main analysis; and (b) to classify mothers on an ordinal measure reflecting the extent of smoking during pregnancy (non-smoker, 1–9 cigs/day, 10+ cigs/day) for use in the supplementary analysis.

### Confounding Factors

To adjust the observed associations between MSDP, *TBR1* and behaviour outcomes for possible confounding by social, family and related factors correlated with MSDP or *TBR1* genotype the following measures were included in the analysis: maternal age at the birth of the child; maternal education at the time of the birth classified in three levels (no formal qualifications, high school qualifications, tertiary qualifications); family SES at the time of the birth classified using the 6-level Elley-Irving scale for New Zealand occupations [77]; the type of family (single parent/two parent) the child entered at birth; a measure of family income based on the average of the family’s gross annual income in each of the first 5 years of the study; a measure of family living standards based on an average of interviewer ratings of the quality of family living standards assessed over the first 5 years of the study; the child’s birthweight.

### Statistical methods

#### Allele grouping

Preliminary examination of the distribution of *TBR1* genotype showed that only 5 participants were homozygous for the minor allele (13-copy repeat). Given the very small number of homozygotes, all carriers of the *TBR1* minor allele were combined into a single group for analysis purposes, referred to as ‘other’. We also used a division of homozygous major allele vs other genotypes for the other three repeats, which replicated published studies on the *AP2-β* loci and was similar to previous work on the *AR* microsatellite, which categorized alleles as “long” vs “short” [24, 58, 60].

#### Associations with microsatellite variants

Associations between microsatellite variants (classified as homozygous major allele vs other) and the measures of child behaviour, cognitive ability, personality/self-esteem and mental health were tested for statistical significance using one way analysis of variance. The strength of each association was summarised by the point biserial correlation between the microsatellite and the outcome. Two way ANOVA models with sex and microsatellite as factors were used to test for sex by microsatellite interactions. A Bonferroni correction was applied to adjust for multiple comparisons. The Bonferroni corrected *p*-value calculated to take into account the average correlations between the 18 outcome measures (r = 0.23) across the four microsatellite variants was *p* = 0.0019.

#### Modelling the TBR1 by MSDP interaction

The joint associations between *TBR1* genotype (homozygous major allele/other) MSDP (no/yes) and antisocial behaviour outcomes (Table 2) were modelled by fitting a series of saturated regression models in which each behavioural outcome was modelled as a function of *TBR1* genotype, MSDP and the multiplicative interaction of *TBR1* and MSDP. For the measures of childhood and adolescent conduct problems multiple linear regression models were fitted; for the count measures of violent/property offences and arrests/convictions, Poisson regression models were fitted with a deviance correction for over-dispersion. These models were then extended to control for potential confounding by social, family and related factors (Table 3). To control for the possibility of Type 1 errors due to multiple significance testing both sets of analyses were supplemented by fitting a multivariate regression model to conduct simultaneous tests of significance of the effects of *TBR1*, MSDP and their interaction. Analyses were also extended to examine the effect of smoking frequency during pregnancy (0, 1–9, 10+ cigs/day) to test for multiplicative interactions with gender and to examine the possible implications of ethnic stratification.

**Table 2:** Associations between microsatellite variants and measures of child behaviour, cognitive ability, personality/self-esteem and mental health (European sample only). The *AP-2β* minisatellite showed no significant association so was excluded from the table

	*AR*-GGN homozygous major vs rest		*AP-2β*-CAAA homozygous major vs rest		*TBR1* homozygous major vs rest	
Measure	*r*	*p*	*r*	*p*	*r*	*p*
Childhood behaviour (7–9 years)	(*N* = 454–459)		(*N* = 582–589)		(*N* = 563–570)	
Conduct problems	0.056	0.23	−0.055	0.18	0.141	0.0007**
Attention problems	0.049	0.29	−0.061	0.14	0.045	0.29
Anxiety/withdrawal	0.019	0.68	−0.039	0.34	0.103	0.015*
Cognitive ability^a^	(*N* = 348–371)		(*N* = 451–482)		(*N* = 442–468)	
WISC-R IQ (8–9 years)	−0.104	0.046*	0.085	0.062	−0.015	0.75
Scholastic ability (13 years)	−0.083	0.12	0.136	0.004*	−0.042	0.38
Personality/self-esteem	(*N* = 428–433)		(*N* = 554–557)		(*N* = 542–544)	
EPI neuroticism (14 years)	0.039	0.41	−0.006	0.88	0.068	0.11
EPI extroversion (14 years)	−0.040	0.41	−0.072	0.08	0.061	0.16
TPI novelty seeking (16 years)	0.017	0.72	−0.045	0.29	0.034	0.43
Self-esteem (15 years)	−0.077	0.11	0.011	0.79	−0.051	0.23
Mental health (SCL-90, 18 years)	(*N* = 453)		(*N* = 588)		(*N* = 568)	
Somatisation	0.060	0.15	−0.024	0.56	0.011	0.80
Anxiety	0.096	0.040*	0.020	0.63	0.022	0.60
Phobic anxiety	0.078	0.10	0.036	0.38	−0.022	0.61
Obsessive compulsive	0.037	0.44	−0.042	0.31	0.036	0.39
Depression	0.037	0.43	−0.009	0.83	0.037	0.38
Interpersonal sensitivity	0.060	0.21	0.012	0.78	0.054	0.20
Psychoticism	0.061	0.19	−0.110	0.008*	0.001	0.97
Paranoid ideation	0.081	0.086	−0.064	0.12	0.046	0.28
Hostility	−0.024	0.60	−0.028	0.49	0.087	0.039*

^a^Cognitive outcomes assessed only for cohort members resident in Christchurch urban region (approximately 80% of total cohort at each age)

*Nominally significant (*p* < 0.05) but didn't withstand Bonferroni correction

**Significant following Bonferroni correction

**Table 3:** Associations between *TBR1* genotype, MSDP and measures of offspring antisocial behaviour

*TBR1* genotype		MSDP	
		No	Yes
(a) Childhood conduct problems (7–9 years)			
Homozygous major allele	Mean (SD)	98.5 (8.4)	99.4 (8.6)
	N	312	140
Other	Mean (SD)	99.2 (9.0)	107.6 (13.2)
	N	79	39
Fitted regression model: main effect *TBR1* (B = 0.71, SE = 1.12, *p* = 0.53); main effect MSDP (B = 0.93, SE = 0.91, *p* = 0.31); *TBR1* x MSDP interaction (B = 7.51, SE = 1.97, *p* < 0.001)			
(b) Adolescent conduct problems (15–16 years)			
Homozygous major allele	Mean (SD)	98.6 (8.8)	100.7 (10.5)
	N	300	136
Other	Mean (SD)	97.8 (6.8)	107.5 (14.1)
	N	75	39
Fitted regression model: main effect *TBR1* (B = -0.85, SE = 1.22, *p* = 0.49); main effect MSDP (B = 2.04, SE = 0.98, *p* = 0.04); *TBR1* x MSDP interaction (B = 7.70, SE = 2.11, *p* < 0.001)			
(c) Self-reported property/violent offences (14–25 years)			
Homozygous major allele	Mean (SD)	9.2 (35.0)	15.1 (52.6)
	N	303	139
Other	Mean (SD)	5.8 (15.3)	26.9 (83.6)
	N	78	36
Fitted regression model: main effect *TBR1* (B =−0.46, SE = 0.34, *p* = 0.18); main effect MSDP (B = 0.50, SE = 0.20, *p* = 0.01); *TBR1* x MSDP interaction (B = 1.03, SE = 0.43, *p* = 0.02)			
(d) Self-reported arrests or convictions (16–25 years)			
Homozygous major allele	Mean (SD)	0.6 (2.1)	1.3 (4.5)
	N	303	139
Other	Mean (SD)	0.3 (0.8)	2.0 (5.4)
	N	78	36
Fitted regression model: main effect *TBR1* (B = −0.83, SE = 0.44, *p* = 0.06); main effect MSDP (B = 0.78, SE = 0.20, *p* < 0.001); *TBR1* x MSDP interaction (B = 1.27, SE = 0.51, *p* = 0.01)			

#### Supplementary analyses

To address the issue that the study findings may have been influenced by selection bias attributable to the processes of sample loss and failure of genotyping, a data weighting strategy was used in which the data were first stratified by socio-demographic characteristics at birth to estimate the probability of inclusion in the analysis sample for each association. Typically this showed the presence of modest but statistically significant (*p* < 0.05) tendencies for some analysis samples to under-represent participants from socioeconomically disadvantaged backgrounds (low parental education, low SES families, single parent families). All data were then re-analysed with each participant weighted by the inverse probability of sample selection. The results for the weighted analyses were negligibly different from results for the unweighted analyses reported in this paper, suggesting that the study findings were unlikely to be affected by selection bias. Finally, the robustness of study findings was checked against re-analysis on the full sample including non-European participants.

## Results

### Association analyses

We examined associations between four short tandem repeats in the *TBR1*, *AR* and *AP-2β* genes (Table 1) and a series of 18 measures of personal characteristics, temperament and behaviours in the CHDS cohort (Table 2). The GGN microsatellite in exon 1 of the *AR* gene showed modest nominally significant associations between a dichotomous (homozygous major allele GGN_23_ carried by 70% of individuals vs rest) measure and two outcomes: child IQ (r = −0.104, *p* = 0.046) and anxiety symptoms (r = 0.096, *p* = 0.040). Those homozygous for the major allele scored higher on IQ and lower on anxiety symptoms. Neither association withstood correction for multiple comparisons. There was no apparent association with neuroticism or extroversion. The CAAA_4-5_ repeat polymorphism in intron 2 of the *AP-2β* gene showed nominally significant correlations with two measures: child scholastic ability (*r* = 0.136, *p* = 0.004) and a measure of psychotic symptoms (*r* = -0.110, *p* = 0.008). Carriers of the minor allele scored higher on scholastic ability at age 13 and lower on psychotic symptoms at age 18. We found no clear associations for the minisatellite in intron 1 of the *AP-2β* gene. The AC_12-13_ microsatellite in the promoter region of the *TBR1* gene was associated with three outcomes. The 22% of individuals who carried the minor allele (AC_13_), scored higher on the measures of childhood conduct problems (*r* = 0.141, *p* = 0.0007), childhood anxiety/withdrawal (*r* = 0.102, *p* = 0.015), and hostility symptoms in adolescence (*r* = 0.087, *p* = 0.039). Only the association with childhood conduct problems withstood correction for multiple comparisons.

Reanalysis of the data including those of non-European ancestry identified a small number of additional nominally significant (*p* < 0.05) associations between the GGN microsatellite and the mental health measures of somatisation, phobic anxiety and psychoticism (*r* = 0.088–0.094), indicative of lower scores on these measures for those who were homozygous for the major allele. The lowest P value for these tests was 0.006, for an association between the homozygous major allele genotype and lower levels of anxiety, and there was nothing significant following Bonferroni correction. Otherwise essentially the same pattern of findings applied to the full sample.

Tests of sex by genotype interaction identified four nominally significant interactions: for *AR* GGN on childhood conduct problems (*p* = 0.013); and for *AP2-β* CAAA on childhood conduct problems (*p* = 0.043), childhood attention problems (*p* = 0.035) and interpersonal sensitivity in adolescence (*p* = 0.035). The associations with the measures of childhood behaviour problems were stronger for males, and the association with interpersonal sensitivity stronger for females. However, none of these interactions withstood Bonferroni correction. No sex interactions were observed for the minisatellite in intron 1 of the *AP-2β* gene or for the *TBR1* microsatellite.

The most significant association we found overall was for the *TBR1* microsatellite minor allele (AC_13_), carried by 22% of the sample, with childhood conduct problems at age 7–9. This was statistically significant after Bonferroni correction for multiple comparisons (r = 0.141, *p* = 0.0007), and we performed no further analyses on the other three repeats. For the reasons outlined above we examined one additional hypothesis: that there may be a joint effect of *TBR1* and MSDP on childhood conduct problems, and we discovered an interaction. We then replicated the analysis by widening the outcomes to other measures of antisocial behaviour. These included measures of adolescent conduct problems (15–16 years), self-reported property/violent offences (14–25 years) and self-reported arrests or convictions (16–25 years).

### Associations between smoking during pregnancy, *TBR1* and subsequent antisocial behaviour

Table 3 shows the sample cross-classified according to history of MSDP (no/yes) and *TBR1* microsatellite genotype (homozygous major allele/other). For each classification the table reports the mean scores on a series of dependent variables representing measures of antisocial behaviour over the life course. The table also reports the results of regression models fitted to the data for each outcome including tests of significance of: a) the main effect of *TBR1*; b) the main effect of MSDP; c) the *TBR1* by MSDP interaction. The table shows that:

1. In three out of four cases there was a significant (*p* < 0.05) main effect of MSDP, reflecting a pervasive association between MSDP and higher rates of subsequent anti-social behaviour assessed up to the age of 25.
2. There were no significant main effects for *TBR1* reflecting the fact that in the absence of MSDP overall rates of antisocial behaviour did not vary with *TBR1*.
3. In all analyses there was a significant (*p* < 0.05) MSDP by *TBR1* interaction, reflecting the fact that the effects of MSDP on antisocial behaviour were more marked for the ‘other’ strata who carried at least one *TBR1* minor allele than for the *TBR1* homozygous major allele group.

As noted in Methods, the analysis in Table 3 is based on a classification of *TBR1* genotype in which all carriers of the *TBR1* minor allele have been classified into a single group. This was done because of the very small number (*n* = 5) who were homozygous for the minor allele. At the same time it is of interest to note that elaboration of the data in Table 3 according to the number of *TBR1* minor alleles showed a pattern of data for three of the four outcomes consistent with an increasing effect of MSDP on antisocial behaviour with an increasing number of *TBR1* minor alleles (see Table S1 in Additional file 1).

A limitation of the analysis in Table 3 is that it reports multiple tests of significance, thus increasing risks of type 1 statistical errors. To address this issue the data in the table were re-analysed using a multivariate regression modelling approach to test the joint significance of effects across all outcomes (see Methods). This analysis confirmed the presence of a just significant main effect of MSDP (F(4,520) = 2.38, *p* = 0.05), the absence of a significant main effect for *TBR1* (F(4,520) = 0.57, *p* = 0.59) and a significant MSDP x *TBR1* interaction (F(4,520) = 3.95, *p* < 0.005).

### Adjustment for confounding factors

To examine the possible effects of confounding social, economic and related factors, the analyses in Table 3 were extended to include a range of covariate factors correlated with MSDP. These factors included measures of: maternal age, maternal education, family socio-economic status, family income, family living standards, family type (single parent/two parent) and birthweight. Table 4 reports tests of the main effects and the *TBR1* by MSDP interaction after covariate adjustment. This table shows that in all cases the significant *TBR1* x MSDP interaction persisted. A multivariate regression model also confirmed the presence of a significant *TBR1* x MSDP interaction (F(4,501) = 3.3, *p* = 0.01). However, the overall main effect of MSDP was no longer significant after covariate adjustment (F(4,501) = 1.64, *p* = 0.16).

**Table 4:** Estimated regression coefficients for the effects of *TBR1* genotype, MSDPand *TBR1* by MSDP interaction after adjustment for confounding

	*TBR1* genotype		MSDP		*TBR1* by MSDP interaction	
Outcome	B(SE)	*p*	B(SE)	*p*	B(SE)	*P*
Conduct problems (7–9 years)	0.84 (1.07)	0.43	−0.26 (0.88)	0.77	6.47 (1.86)	<0.001
Conduct problems (15–16 years)	−0.61 (1.19)	0.61	0.83 (0.97)	0.40	6.23 (2.04)	< 0.005
Property/violent offences (14–25 years)	−0.91 (0.47)	0.05	0.65 (0.21)	< 0.005	1.24 (0.54)	0.02
Arrests/convictions (16–25 years)	−0.32 (0.42)	0.45	0.76 (0.24)	< 0.005	2.21 (0.49)	< 0.001

### The effect of frequency of smoking

To take account of variations in MSDP the data were re-analysed using an ordered categorical measure of MSDP: non-smoker; 1–9 cigarettes per day; 10+ cigarettes per day. This analysis produced the same overall pattern of findings as those reported in Tables 3 and 4. After covariate adjustment the multivariate regression model again showed a significant main effect for MSDP (F(4,501) = 2.51, *p* = 0.04) and a significant *TBR1* by MSDP interaction (F(4,501) = 2.85, *p* = 0.02).

### The effect of gender

To examine the extent to which the findings may be moderated by gender, the analyses were extended to include gender as a further factor and tests of gender x *TBR1* x MSDP interaction were conducted. No significant three-way interactions were found, suggesting that the *TBR1* x MSDP interaction was evident for both males and females.

### The effect of ethnic stratification

To examine the implications of ethnic stratification, the data were re-analysed including an additional 91 participants with data on *TBR1*, MSDP and behaviour outcomes who were of non-European (Maori or Pacific Island, by self-definition) ethnic origin. The fitted regression models were extended to incorporate ethnicity as a factor and to test for ethnicity by *TBR1* by MSDP interactions. While the analysis lacked statistical power to draw strong conclusions, for all outcomes the *TBR1* by MSDP interaction appeared to be somewhat weaker for non-European sample members. This was reflected in the multivariate regression model which showed that, after covariate adjustment, there was a significant ethnicity by *TBR1* by MSDP interaction (F(4,577) = 2.62, *p* = 0.034), in addition to the existing *TBR1* by MSDP interaction (F(4,577) = 3.03, *p* = 0.017).

Collectively the results in Tables 3 and 4 and the additional analyses reported above confirm the presence of a robust and persistent *TBR1* by MSDP interaction in which MSDP had greater effects on the risks of subsequent antisocial behaviours for those with the “other” genotype who carried at least one copy of the *TBR1* minor allele when compared with those who were homozygous for the major allele. These findings held after: a) control for covariate factors; b) variation in the measurement of MSDP; c) tests of gender interaction. This interaction appeared to be stronger amongst those of European ancestry.

### Linkage disequilibrium with nearby single nucleotide polymorphisms

Single nucleotide polymorphisms (SNPs) in our cohort had been genotyped as part of a genome wide association study [78]. We examined all SNPs within 150 k bases (kb) of *TBR1* to see if any of them could explain the effects of the microsatellite. Three SNPs located 37-64 kb upstream of the gene, rs3769963, rs1116173 and rs6727917, are in at least partial linkage disequilibrium with the microsatellite polymorphism. We analysed these SNPs individually and jointly with microsatellite genotype. Individually all three SNPs showed evidence of a gene x MSDP interaction on childhood conduct problems (data not shown). Of the three, rs3769963 showed the strongest evidence of interaction and produced results that were closest to those for the microsatellite. However, in all cases the R squared values for these models (0.032-0.042) were substantially less than the R-squared value for the microsatellite model (0.058). Also, rs3769963 is located 65 kb upstream of the *TBR1* gene’s transcriptional start site, and while this is not inconsistent with a regulatory role, its level of LD with the promoter microsatellite polymorphism was 2.6 × higher than that of any other SNP within 150 kb of *TBR1*. In contrast, the levels of LD between the microsatellite and its neighbouring SNPs were very low (R-squared < 0.0005 and D’ < 0.2).

## Discussion

Our main finding was that the minor allele of the AC_12-13_ microsatellite in the upstream promoter region of the *TBR1* gene interacts with MSDP to increase risk of antisocial behaviour, a putative gene by environment (G x E) interaction which remained significant after adjustment for potentially confounding social and contextual factors, gender and frequency of smoking. We found some evidence that the interaction may be stronger in individuals of European descent, but the number of non-Europeans in our cohort was too small to rule out a stochastic effect.

We also report results for two intronic repeats in the *AP-2β* transcription factor gene, and a GGN microsatellite in the *AR* gene’s first exon. All of these have previously been associated with personality or behaviour-related phenotypes [24, 54–61]. Our finding that the most common allele of the *AR* microsatellite, GGN_23_, was associated with fewer anxiety-related symptoms was consistent with previous work [24], though in our study this wasn’t significant following correction for multiple hypotheses. GGN_23_, elsewhere referred to as GGC_16_ [54], is the shorter of the two most common alleles and has been associated with reduced [51] and increased [52] activation of androgen receptor protein in different systems. Our only nominally significant results for the two previously studied *AP-2β* repeats were that carriers of the minor allele of the CAAA_4-5_ microsatellite polymorphism scored lower on psychotic symptoms at age 18 and higher on scholastic ability at age 13. The former was unexpected in view of a previous study reporting higher rates of anxiety and related traits in carriers of this allele among 137 Caucasians living in Sweden [60]. However, none of our findings in relation to this repeat were significant following Bonferroni correction.

We note that evidence for common genetic risk factors for many psychiatric, substance abuse and conduct disorder phenotypes [79] suggests that the lack of consistent associations in our data may indicate false positives. This argument does not apply to the *TBR1* interaction, which did replicate across different ages and measures, but because this study is the first to link the *TBR1* gene with antisocial behaviour, or with the effects of MSDP, the results should be treated with caution until replication can be attempted in another cohort. This point has been highlighted by a meta-analysis showing that first reports of gene-environment interactions are often not confirmed by studies attempting replication, presumably due to under-reporting of negative results [80]. Several considerations do, however, support the validity of our results at this stage. Firstly, our interaction hypothesis emerged from a coincidence of rare interests in conserved microsatellites, their influence on the genetics of behaviour, and the effects of MSDP. Previous attempts at finding the interaction are therefore very unlikely, and we have presented all of our negative results here. Furthermore, our sample size is substantial by the standards of G x E interaction studies [80], and the P-value of the interaction we observed also compares favourably with previous reports of G x E interaction involving MSDP and behavioural or cognitive phenotypes [81–88]. In this field, high quality, multi-method phenotype measurement similar to that used for this study is usually performed. Along with adjustment for confounding factors this provides some compensation for sample sizes being smaller than most contemporary complex trait GWAS, which are thought to be limited by insufficiently detailed phenotyping [89].

The association between *TBR1*, MSDP and antisocial behaviour may be due to modification of the effects of tobacco exposure on embryonic development by *TBR1* genotype. G x E interaction involving MSDP has been demonstrated directly in knockout mice [90], and both MSDP and microsatellite polymorphism have been linked to epigenetic modifications in offspring [12, 91, 92], However, the Genotype-Tissue Expression Project has so far not identified expression quantitative trait loci for *TBR1* [93]. An alternative to the G x E interaction explanation is suggested by the likely existence of genetic factors which predispose mothers to both pregnancy smoking and antisocial behaviour [94]. An important role for gene-gene interaction (epistasis) would be consistent with large scale studies showing that siblings who are differentially exposed to prenatal tobacco are similarly prone to antisocial behaviour [95, 96]. Indeed, it has been suggested that promoter-associated and exonic microsatellites may be particularly likely to participate in epistasis due to their interactions with *trans*-acting factors [11].

SNPs associated with *TBR1* have not been identified by GWAS of antisocial behaviour or related phenotypes [5–8]. Nevertheless, the possibility that effects of the microsatellite polymorphism were not detectable by these GWAS is supported by its very low level of LD with neighbouring SNPs, and by the absence of its effect on antisocial behaviour in individuals whose mothers didn’t smoke during pregnancy. The association we report, and the extremely high level of conservation of the *TBR1* microsatellite [34], suggest that its putative functional importance deserves further testing. One possible approach would be to investigate the effect on transcriptional frequency of each allele using reporter plasmids in cultured neuronal cells [97]. Such assays have been used in previous studies to show that changes in repeat number of promoter-associated poly-AC microsatellites can affect frequency of transcription [98, 99]. However it is notable that they don’t necessarily provide an accurate model of gene expression in developing brain, and functional studies have shown cell type dependent microsatellite length effects, in one case with opposite effects in two different cell types [100].

## Conclusions

We found limited support for the previously observed association between the *AR* gene’s exonic GGN microsatellite polymorphism and anxiety-related symptoms, but we found no significant results for two previously studied repeats in the *AP2-β* transcription factor gene. Our findings in relation to the AC microsatellite in the *TBR1* promoter region suggest that it deserves further investigation, including replication in a separate cohort of its associations with antisocial behaviour and MSDP. They also demonstrate the potential value of investigating the functional importance of conserved microsatellites in general.

## Additional file

Additional file 1: Supplementary methods and Table S1. (DOCX 20 kb)
