# Supplementary material for: Microsatellite polymorphisms associated with human behavioural and psychological phenotypes including a gene-environment interaction
Source: BMC Med Genet. 2017 Feb 3;18:12. doi: 10.1186/s12881-017-0374-y (PMC5291968; doi:10.1186/s12881-017-0374-y)
Supplement: Additional file 1: — Supplementary methods and Table S1. (DOCX 20 kb) [file 12881_2017_374_MOESM1_ESM.docx]

**Description of Measures**

A series of 18 measures were selected from the study database to examine associations with the microsatellite variants. These measures spanned the following domains:

**Child behaviour (7-9 years)**

When sample members were aged 7, 8 and 9 years parental and teacher reports of child behaviour problems were obtained using an instrument that combined items from the Rutter [1] and Conners [2, 3] parent and teacher questionnaires. Selected items from these scales were used to derive scale measures of child behaviour problems in each of three domains [4, 5]:

*Child conduct problems* assessed the extent to which the child displayed conduct disordered or oppositional behaviours. Selected items spanned a range of problems relating to disobedience and defiance of authority, fits of temper and irritability, aggression or cruelty towards others, destruction of property, lying, stealing and other related behaviours.

*Child attentional problems*assessed the extent to which the child displayed inattentive/hyperactive behaviours. Selected items spanned a range of symptoms relating to inattentive behaviour, short attention span, distractibility, restlessness and hyperactivity.

*Child anxiety/withdrawal* assessed the extent to which the child displayed symptoms relating to shy, anxious or withdrawn behaviours.

All items were scored on a 3-point scale ranging from ‘not at all’ to ‘a great deal’. Confirmatory factor analysis of the selected items for each domain and each source (parents, teachers) showed that, in each case, the items could be scaled as unidimensional scales representing the extent of child conduct problems, attentional problems or anxiety/withdrawal as reported by parents and teachers [4, 5]. Scale scores representing the extent of behaviour problems in each domain were created by summing the parent and teacher item scores at each age. These scale scores were then averaged over the interval from 7-9 years to provide overall measures reflecting the severity of conduct problems, attentional problems and anxiety/withdrawal in middle childhood. The reliabilities of these scales, assessed using coefficient α, were 0.97 (conduct problems), 0.93 (attentional problems, 0.87 (anxiety/withdrawal).

**Cognitive ability**

*Child IQ (8-9 years).* Child cognitive ability was assessed at ages 8 and 9 years using the Revised Wechsler Intelligence Scale for Children (WISC-R) [6]. At each age, children were administered four verbal subscales (information, similarities, arithmetic, vocabulary) and four performance subscales (picture completion, block design, object assembly, coding). The scaled subtest scores were then prorated to provide measures of verbal IQ, performance IQ and total IQ at ages 8 and 9. The total IQ scores were used in the present analysis. The reliabilities of these scores, assessed using split half measures, were 0.93 at age 8 and 0.95 at age 9. For the purposes of this analysis the observed WISC-R total IQ scores at age 8 and 9 years were combined by averaging over the two assessments.

*Scholastic ability (13 years)*. At age 13 participants completed the Test of Scholastic Ability (TOSCA) [7]. This test assesses the extent to which individuals possess the skills and competencies required for academic work at high school. The test was scored as described in the test manual to provide a total TOSCA score. The reliability of this test, measured using coefficient α, was 0.95.

Due to resource constraints cognitive testing was conducted only for cohort members who were resident in the Christchurch urban region at the time of assessment. This sample represented approximately 80% of the total cohort assessed at each age.

**Personality and self-esteem**

*Neuroticism and Extroversion (14 years)*.Child personality was assessed at age 14 years using the short form of the Eysenck Personality Inventory (EPI) [8]. This measure comprises two subscales of neuroticism (10 items) and extroversion (10 items). Items were scored on a 3-point scale (not like me/ a bit like me/ a lot like me). Total scale scores for neuroticism and extroversion were constructed based on a sum of the items in each subscale, scored such that higher scores implied higher neuroticism or extroversion. The two scales were of moderate internal consistency (α = 0.80, 0.83 respectively).

*Novelty seeking (16 years).*When sample members were aged 16 years they were administered the novelty seeking items of the Tridimensional Personality Questionnaire [9].These items were summed to produce an overall novelty seeking measure. The scale was of moderate internal consistency (α = 0.76).

*Self-esteem (15 years).* Self-esteem was assessed at age 15 using the global scale of the Coopersmith Self-Esteem Inventory [10] obtained by summing of the four subscale scores (general, academic, social, and home). The global scale score had high internal consistency (α = 0.87).

**Mental health problems**

At age 18 participants were interviewed concerning aspects of their mental health. As part of this interview, participants were questioned on current (over the past month) mental health symptomatology using the Symptom Checklist 90 (SCL-90) [11]. This scale assesses mental health symptoms in nine sub-scales: *somatisation; anxiety; phobic anxiety; obsessive-compulsive; depression; interpersonal sensitivity; psychoticism; paranoid ideation; and hostility*. Sub-scale scores were calculated from a sum of the items in each domain. Scales were of moderate to good internal consistency, with αvalues ranging from 0.64 to 0.87.

Table S1. Associations between *TBR1* genotype (number of minor alleles), maternal smoking during pregnancy and measures of offspring antisocial behaviour.

(a) Childhood conduct problems (7-9 years)

|  |  | Pregnancy smoking | |
| --- | --- | --- | --- |
| *TBR1* number of minor alleles |  | No | Yes |
| 0 | Mean (SD) | 98.5 (8.4) | 99.4 (8.6) |
|  | N | 312 | 140 |
| 1 | Mean (SD) | 99.4 (9.1) | 107.3 (12.8) |
|  | N | 76 | 37 |
| 2 | Mean (SD) | 94.7 (7.6) | 113.7 (24.4) |
|  | N | 3 | 2 |

Fitted regression model:main effect *TBR1* (B = 0.46, SE = 1.06, p = 0.66); main effect pregnancy smoking (B = 0.90, SE = 0.90, p = 0.32); *TBR1* x pregnancy smoking interaction (B = 7.28, SE = 1.83, p < 0.001)

(b) Adolescent conduct problems (15-16 years)

|  |  | Pregnancy smoking | |
| --- | --- | --- | --- |
| *TBR1* number of minor alleles |  | No | Yes |
| 0 | Mean (SD) | 98.6 (8.8) | 100.7 (10.5) |
|  | N | 300 | 136 |
| 1 | Mean (SD) | 97.7 (6.9) | 107.2 (14.2) |
|  | N | 72 | 37 |
| 2 | Mean (SD) | 98.7 (5.1) | 113.1 (17.4) |
|  | N | 3 | 2 |

Fitted regression model: main effect *TBR1* (B = -0.74, SE = 1.15, p = 0.52); main effect pregnancy smoking (B = 2.06, SE = 0.97, p = 0.03); *TBR1* x pregnancy smoking interaction (B = 7.22, SE = 1.96, p < 0.001)

(c) Self-reported property/violent offences (14-25 years)

|  |  | Pregnancy Smoking | |
| --- | --- | --- | --- |
| *TBR1* number of minor alleles |  | No | Yes |
| 0 | Mean (SD) | 9.2 (35.0) | 15.1 (52.6) |
|  | N | 303 | 139 |
| 1 | Mean (SD) | 6.0 (15.6) | 25.9 (84.6) |
|  | N | 75 | 35 |
| 2 | Mean (SD) | 1.3 (1.5) | 64.0 (-) |
|  | N | 3 | 1 |

Fitted regression model: main effect *TBR1* (B = -0.46, SE = 0.33, p = 0.17); main effect pregnancy smoking (B = 0.49, SE = 0.19, p = 0.01); *TBR1* x pregnancy smoking interaction (B = 1.04, SE = 0.41, p = 0.01)

(d) Self-reported arrests or convictions (16-25 years)

|  |  | Pregnancy smoking | |
| --- | --- | --- | --- |
| *TBR1* number of minor alleles |  | No | Yes |
| 0 | Mean (SD) | 0.6 (2.1) | 1.3 (4.5) |
|  | N | 303 | 139 |
| 1 | Mean (SD) | 0.3 (0.8) | 2.1 (5.4) |
|  | N | 75 | 35 |
| 2 | Mean (SD) | 0.0 (-) | 0.0 (-) |
|  | N | 3 | 1 |

Fitted regression model: Main effect *TBR1* (B = -0.83, SE = 0.43, p = 0.05); Main effect pregnancy smoking (B = 0.80, SE = 0.19, p < 0.001); *TBR1* x pregnancy smoking interaction (B = 1.20, SE = 0.49, p = 0.01)

**References**

1. Rutter, M., J. Tizard, and K. Whitmore, *Education, health, and behaviour; psychological and medical study of childhood development*. 1970, New York: Wiley.

2. Conners, C.K., *Symptom Patterns in Hyperkinetic, Neurotic, and Normal Children.* Child Development, 1970. **41**(3): p. 667-682.

3. Conners, C.K., *A Teacher Rating Scale for Use in Drug Studies with Children.* American Journal of Psychiatry, 1969. **126**(6): p. 884–888.

4. Fergusson, D.M. and L.J. Horwood, *The Structure, Stability and Correlations of the Trait Components of Conduct Disorder, Attention Deficit and Anxiety/Withdrawal Reports.* Journal of Child Psychology & Psychiatry & Allied Disciplines, 1993. **34**(5): p. 749-766.

5. Fergusson, D.M., L.J. Horwood, and M. Lloyd, *Confirmatory factor models of attention deficit and conduct disorder.* Journal of Child Psychology and Psychiatry, 1991. **32**(2): p. 257-274.

6. Wechsler, D., *Manual for the Wechsler Intelligence Scale for Children - Revised*. 1974, New York: Psychological Corporation.

7. Reid, N.A., et al., *Test of Scholastic Abilities*. 1981, Wellington: Ministry of Education.

8. Eysenck, H., *Manual of the Eysenck Personality Inventory*. 1964, London: London University Press.

9. Cloninger, C.R., *A systematic method for clinical description and classification of personality variants.* Archives of General Psychiatry, 1987. **44**: p. 573-588.

10. Coopersmith, S., *SEI - Self Esteem Inventories*. 1981, Palo Alto, CA: Consulting Psychologists Press.

11. Derogatis, L.R., R.S. Lipman, and L. Covi, *SCL-90: an outpatient psychiatric rating scale - preliminary report.* Psychopharmacol Bulletin, 1973. **9**(1): p. 13-28.
